# An anomalous left coronary artery with a malignant course: coronary angiography and myocardial perfusion imaging with computed tomography

**DOI:** 10.1007/s12471-015-0788-0

**Published:** 2016-01-07

**Authors:** M. A. de Graaf, A. R. van Rosendael, L. J. Kroft, H. W. Vliegen, M. G. Hazekamp, J. J. Bax, A. J. Scholte

**Affiliations:** 1 Department of Cardiology, Leiden University Medical Center, Albinusdreef 2, 2333 ZA Leiden, The Netherlands; 2 The Interuniversity Cardiology Institute of the Netherlands, Utrecht, The Netherlands; 3 Department of Radiology, Leiden University Medical Center, Leiden, The Netherlands; 4 Department of Cardiothoracic Surgery, Leiden University Medical Center, Leiden, The Netherlands

A 41-year old woman presented at the emergency department because of collapse during running. Coronary computed tomography angiography (CTA) showed no atherosclerosis (Fig. 1). However, as demonstrated by the double oblique view of the sinus of Valsalva in Panel A and 3D-rendering of Panel B, the left coronary artery (LCA) originated from the right coronary sinus. The LCA has an acute angle and a ‘slitlike’ ostium which can collapse in a valve-like manner during exercise. The incidence of this finding on CTA ranges from 0.7– 6.6 % [1, 2]. Adenosine stress CT myocardial perfusion imaging (CTP) was performed. Panel C demonstrates a sub-endocardial perfusion defect in the LCA region (white arrows). Panel D represents the polar map of the transmural perfusion ratio. Surgical unroofing was performed successfully and no further events have occurred [3]. In patients presenting with collapse a malignant coronary anomaly can be observed. CTP can subsequently be performed to detect myocardial ischaemia.

## Disclosures

Michiel A. de Graaf is supported by a research grant from the Interuniversity Cardiology Institute of the Netherlands (ICIN, Utrecht, the Netherlands). The Department of Cardiology received research grants from Biotronik, Medtronic, Boston Scientific Corporation, St Jude Medical, Lantheus Medical Imaging and GE Healthcare.

**Fig. 1:**
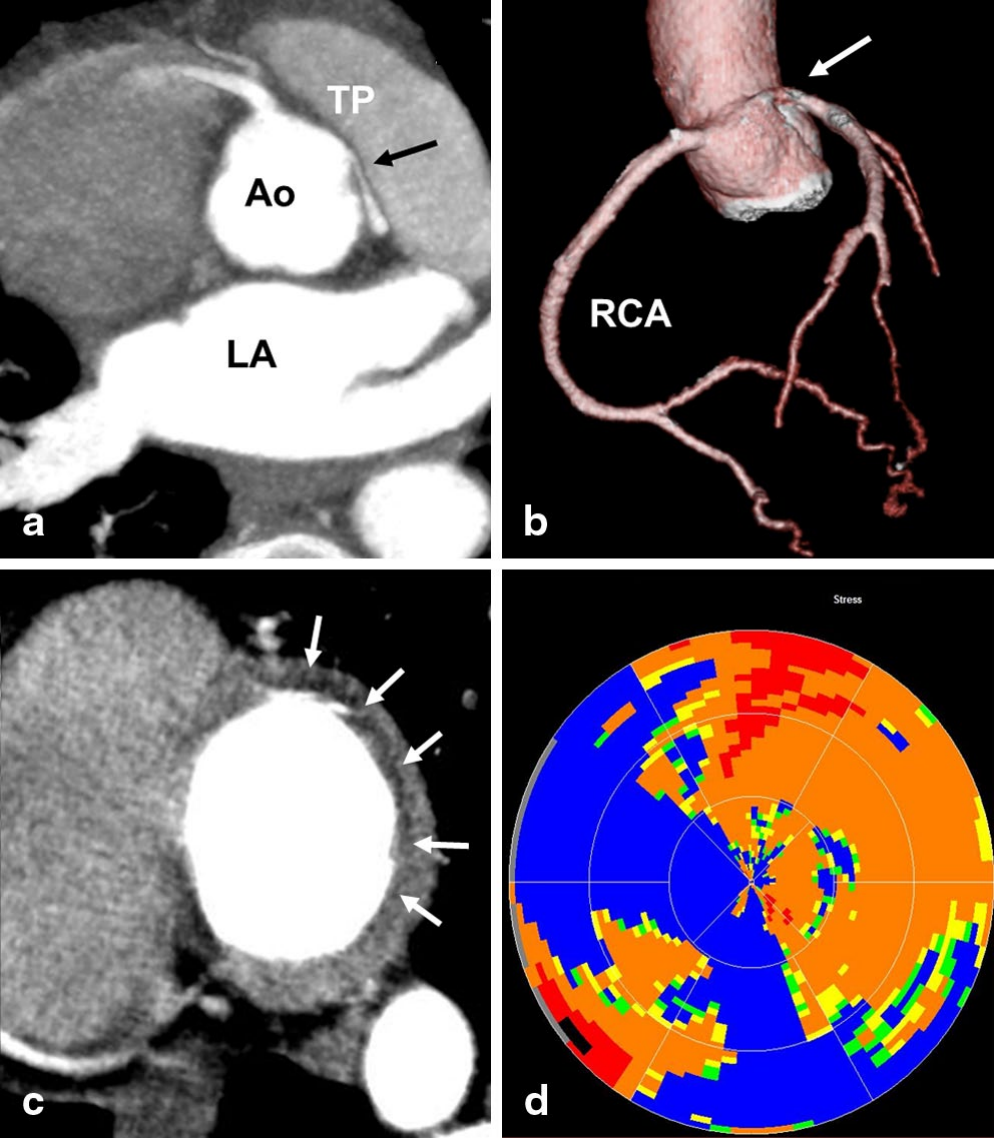
Computed tomography coronary angiography and myocardial perfusion imaging

## OPTIONAL

**Video 1. CT coronary anatomy − 3D-volume rendering**
3D-volume rendered CT coronary angiography demonstrating the aberrant left coronary artery origination from the right coronary sinus. (AVI 59,140 kb)

**Video 2. CTA coronary anatomy - axial cross-section**
Image run through the axial CTA images from cranial to caudal demonstrating the coronary anatomy in relation to the other myocardial structures. The left coronary artery originates from the right coronary sinus and has an acute angle and a ‘slitlike’ ostium. (AVI 67,589 kb)

**Video 3. CT stress myocardial perfusion imaging - short axis**
Double-oblique short axis reconstruction of the stress myocardial perfusion dataset from basal to apical level. A sub-endocardial myocardial perfusion defect is observed in the (antero)lateral wall. Hypo-enhancement in the inferoseptal region was interpreted as artefact. (AVI 76,805 kb)
